# Tandem mass tag-based quantitative proteomics analyses of a chicken-original virulent and its attenuated *Histomonas meleagridis* strain in China

**DOI:** 10.3389/fvets.2023.1106807

**Published:** 2023-03-17

**Authors:** Qiao-Guang Chen, Yu-Ming Zhang, Chen Chen, Shuang Wang, Zai-Fan Li, Zhao-Feng Hou, Dan-Dan Liu, Jian-Ping Tao, Jin-Jun Xu

**Affiliations:** ^1^College of Veterinary Medicine, Yangzhou University, Yangzhou, China; ^2^Jiangsu Co-innovation Center for Prevention and Control of Important Animal Infectious Diseases and Zoonosis, Yangzhou, China

**Keywords:** *Histomonas meleagridis*, virulence, attenuation, TMT-based quantitative proteomic analysis, parallel reaction monitoring

## Abstract

**Introduction:**

*Histomonas meleagridis* can cause histomonosis in poultry. Due to the prohibition of effective drugs, the prevention and treatment of the disease requires new strategies. Questions about its pathogenic mechanisms and virulence factors remain puzzling.

**Methods:**

To address these issues, a tandem mass tag (TMT) comparative proteomic analysis of a virulent strain and its attenuated strain of Chinese chicken-origin was performed.

**Results:**

A total of 3,494 proteins were identified in the experiment, of which 745 proteins were differentially expressed (fold change ≥1.2 or ≤0.83 and *p* < 0.05), with 192 up-regulated proteins and 553 down-regulated proteins in the virulent strain relative to the attenuated strain.

**Discussion:**

Surface protein BspA like, digestive cysteine proteinase, actin, and GH family 25 lysozyme were noted among the proteins up regulated in virulent strains, and these several proteins may be directly related to the pathogenic capacity of the histomonad. Ferredoxin, 60S ribosomal protein L6, 40S ribosomal protein S3, and NADP-dependent malic enzyme which associated with biosynthesis and metabolism were also noted, which have the potential to be new drug targets. The up-regulation of alpha-amylase, ras-like protein 1, ras-like protein 2, and involucrin in attenuated strains helps to understand how it is adapted to the long-term *in vitro* culture environment. The above results provide some candidate protein-coding genes for further functional verification, which will help to understand the molecular mechanism of pathogenicity and attenuation of *H. meleagridis* more comprehensively.

## Introduction

Histomonosis (histomoniasis) is a parasitic disease caused by *Histomonas meleagridis*, also known as ‘blackhead’ or enterohepatitis (1). It can cause serious harm to the turkey breeding industry (2). At present, this disease is more common in free-range chickens, special birds, and zoo birds (2, 3). *Histomonas meleagridis* mainly causes cecitis, hepatitis, and peritonitis in clinical practice (4). After infection, thickening and congestion of the cecal wall first appear. As the disease progresses, the liver appears swollen, with a large number of crater-like necrosis on the surface. In turkeys, mortality from histomoniasis sometimes approaches 100% of a flock (2). In chickens, the incidence is usually 10–30%, up to 50.8%, and the mortality rate is 10–20%, which is equally harmful (5, 6). Negative effects on birds performance may include poor flock uniformity, delayed egg production, and decreased egg production and quality (7). The disease has a high incidence which lead to serious economic losses.

In recent years, histomonosis has re-emerged due to the prohibition of effective anti-histomonad drugs and the increasing popularity of free-range poultry farming (6, 8, 9). The good news is that the establishment of a culture method for *H. meleagridis* and its stable attenuation after multiple passages *in vitro* has opened up a new way for the prevention and control of histomonosis. In this method, the mixed bacterial components of *H. meleagridis* were used to reproduce *in vitro* to reduce the virulence of parasites and prepare live attenuated vaccination. Among the known prevention and therapeutic approaches that have been considered against *H. meleagridis*, live attenuated vaccination was recognized to be the most promising (10). The further development of mono-eukaryotic culture with *Escherichia coli* DH5α instead of mixed flora was of great significance for more subsequent molecular studies (11).

Although its importance to the health of poultry, little is known about the molecular biology and attenuated molecular mechanisms of this parasite. Only in recent years, a small amount of molecular research has appeared. First, the phylogenetic relationship of *H. meleagridis* was determined and the existing sequences were enriched (12). Subsequently the virulent and attenuated parasites were sequenced by *de novo* transcriptome, which further updated the data of *H. meleagridis* (13). About 17 out of 19 proteins further identified by non-comparative two-dimensional electrophoresis (2-DE) of *H. meleagridis* were actin (14). Recent study shown that using two dimensional electrophoresis (2-DE) method, differential proteome analysis of virulent and attenuated turkey-original *H. meleagridis* showed that there were 119 differential protein spots, of which 62 belonged to the virulent and 57 belonged to the attenuated culture (15). In another study, two-dimensional electrophoresis and MALDI-TOF/TOF comparative proteomic analysis were performed on the *in vitro* cultivated virulent and attenuated histomonad parasite. A total of 49 differential proteins were identified, of which 37 belonged to the virulent parasite and 12 to the attenuated parasite (16). However, there are no reports of chicken-original *H. meleagridis*.

Our group obtained the RNA *de novo* assembly of genomic data from chicken-original *H. meleagridis* was obtained (BioProject:PRJNA935845). Data were mapped to NR, NT, Swiss-Prot, KEGG, and KOG database by Blast software to obtain isoform annotations (17). In this study, the protein profiles of virulent and attenuated chicken-original *H. meleagridis* was systematically studied by applying tandem mass tag (TMT)-LC-MS/MS proteomic strategy based on a previous preparation, and some candidate proteins were verified by the targeted parallel reaction monitoring (PRM) method (18, 19). This study contributes to a more detailed understanding of the proteomic differences between the virulent and attenuated strains.

## Materials and methods

### Isolates of *H. meleagridis*

The case came from more than 120 60-day-old *Sanhuang* chickens raised by a farmer in Guangling District, Yangzhou City, Jiangsu Province. On 19 April 2018, the flocks presented with symptoms including crouched head, drooping wings, and excretion of watery greenish yellow diarrhea. Necropsy of the dead chicken showed essentially the same symptoms, with ulcerated lesions in the liver with rounded depressions on the surface, a distinctive crater-like shape. The ceca were swollen and contained casein-like emboli.

Approximately 1 g of necrotic tissues from diseased chicken livers were aseptically removed and placed into a sterile 1.5 ml Eppendorf tubes. The tissues were smashed and inoculated into 10 ml medium that was preheated to 40°C, and then incubated anaerobically in an incubator at 40°C. The medium consisted of 9 ml of M199 medium (Gibco, California, USA), 1 ml of inactivated horse serum (Gibco), and 11 mg of rice flour (Sigma, St. Louis, MO, USA), and was inoculated with a ring of cecal bacteria grown on a Columbia blood agar plate. The cecal bacteria were obtained by inoculating Colombia blood agar plate with the cecal contents of healthy chickens and placing them overnight at 37°C. Cecal bacterial species were identified as *Escherichia coli* and *Klebsiella pneumoniae* by microbial mass spectrometry (MALDI Biotyper).

### Cloning of *H. meleagridis* parasites

The cloning was carried out using the limiting dilution method of monoclonal antibodies. The cultured parasites reaching their growth peak were diluted to ~10^3^ cells/ml with preheated medium, and 1–2 μl of the medium was drawn onto cover slips to observe the parasites under a microscope. Single parasites were labeled and transferred to Eppendorf tubes containing 1 ml of medium and anaerobically cultured in an incubator at 40°C. After 3–4 days of cultivation, the culture broth containing propagated parasites was inoculated into fresh basal medium at a 1:10 ratio for the propagation of the parasites. The cloned parasite strain was named HM-JSYZ-D and cryopreserved.

### Passaging and animal inoculation test of *H. meleagridis*

The *in vitro* passage medium constituted of Medium 199 (Gibco, California, USA) and 10% inactivated horse serum (Gibco), 11 mg of sterilized rice flour (Sigma-Aldrich, St. Louis, MO, USA), with a total volume of 10 ml. Parasites were passaged every 3 days. Long term preservation use 10% dimethyl sulfoxide (Sigma-Aldrich, ShangHai, China) as cryoprotectant. *In vitro, H. meleagridis* with 10 passages was labeled as D10, and *H. meleagridis* with 168 passages was labeled as D168.

To assess whether *H. meleagridis* D168 strain has been attenuated. Thirty *sanhaung* chickens (purchased from the Institute of Poultry, Chinese Academy of Agricultural Sciences, Jiangsu, China) reared in steel cages with wire flooring under specific-pathogen-free conditions were randomly divided into three groups at 14 days of age: D168, D10, NC (10 chickens per group). These chickens were raised in an isolated environment after hatching and fed a drug-free feed formula with free access to water. Animal care and all experiments were performed according to the rules of the Animal Experiment Ethic Committee of Yangzhou University. On the day of grouping, the chickens in group D168 were inoculated cloacally at doses of 2 × 10^5^ JSYZ-D168 cells/chicken; the chickens in group D10 were inoculated cloacally at doses of 2 × 10^5^ JSYZ-D10 cells/chicken; the chickens in group NC served as negative control (unchallenged with virulent strain). On day 28 (14 days post challenge), all remaining chicks were euthanized and observed for cecal and hepatic lesions.

### Collection of virulent and attenuated *H. meleagridis* parasites

The D10 and D168 generations of parasites passaged in the same batch as the animal test were collected and designated as *H. meleagridis*/chicken/China/JSYZ-D/D10 and *H. meleagridis*/chicken/China/JSYZ-D/D168. Each sample was collected in triplicate, and the quality and quantity of *H. meleagridis* was assessed under a microscope using Trypan Blue Stain (TaKaRa, BeiJing, China), ensuring that each sample is no less than 1 × 10^7^ number of parasites. A purification method of continuous washing and centrifugation were used to collect *H. meleagridis* cells and remove most of the bacteria (15). Cultures were concentrated and transferred to 1.5 ml Eppendorf tubes, and histomonads were collected by centrifugation at 200 g for 5 min at 4°C. The bacteria enriched supernatant was discarded and the histomonads were resuspended in M199 medium at 4°C. This step was repeated for three times. The histomonads were finally centrifuged at 8,000 g for 2 min at 4°C, and the precipitate was collected after discarding the supernatant. The collection parasites were stored at −196°C before further use.

### Protein extraction and TMT labeling of parasites

Parasite samples was sonicated three times on ice using a high intensity ultrasonic processor (Scientz) in lysis buffer (8 M Urea, 1% Protease Inhibitor Cocktail). The remaining debris was removed by centrifugation at 12,000 g at 4°C for 10 min. The supernatant was collected and the protein concentration was determined with BCA (Beyotime, HangZhou, China) kit according to the manufacturer's instructions. Equal amounts of each sample protein were taken for enzymatic lysis, appropriate amounts of standard protein were added, the volume was adjusted to a consistent volume with the lysis solution, the final concentration of 20% TCA was slowly added, vortexed, and precipitated at 4°C for 2 h. Centrifuged at 4,500 g for 5 min, discard the supernatant, and wash the sample 2–3 times with pre chilled acetone. After air drying the sample was added to a final concentration of 200 mM TEAB (Sigma, HangZhou, China), the sample was disrupted by sonication. Trypsin (Promega, HangZhou, China) was added at a 1:50 ratio and enzymatically digested overnight at 4°C. Protein solution was reduced with 5 mM dithiothreitol for 30 min at 56°C and alkylated with 11 mM iodoacetamide for 15 min at room temperature in darkness. After trypsin digestion, peptide was desalted by Strata X C18 SPE column (Phenomenex) and vacuum-dried. Peptide was reconstituted in 0.5 M TEAB and processed according to the manufacturer's protocol for TMT kit (Thermo, HangZhou, China). Briefly, one unit of TMT reagent were thawed and reconstituted in acetonitrile. The peptide mixtures were then incubated for 2 h at room temperature and pooled, desalted, and dried by vacuum centrifugation.

### LC-MS/MS analysis

Peptides were separated after dissolved them in solvent A (0.1% formic acid in 2% acetonitrile). The gradient was comprised of an increase from 7% to 25% solvent B (0.1% formic acid in 90% acetonitrile) over 24 min, 25% to 35% in 8 min and climbing to 80% in 4 min then holding at 80% for the last 4 min, all at a constant flow rate of 500 nl/min on an EASY-nLC 1000 UPLC system.

Peptides were injected into the NSI ion source for ionization after separation via the ultra-high-performance liquid phase system and then subjected to Q Exactive Plus mass spectrometry for analysis. The ion source voltage was set to 2.1 kV, and both peptide parent ions and their secondary fragments were detected and analyzed using a high-resolution Orbitrap. The primary mass spectrometry scan range was set to 400–1,500 m/z, and the scan resolution was set to 70,000. The secondary MS scan range was then fixed to a starting point of 100 m/z, and the secondary scan resolution was set to 17,500. Data acquisition mode used a data dependent scanning program whereby the top 20 peptide parent ions with the highest signal intensity were selected after the primary scan to sequentially enter the higher energy collision-induced dissociation (HCD) collision cell for fragmentation using 28% fragmentation energy, again followed sequentially by secondary mass spectrometry. To improve the effective utilization of the mass spectra, the automatic gain control (AGC) was set to 5E4, the signal threshold was set to 78,000 ions/s, the maximum injection time was set to 64 MS, and the dynamic exclusion time of the tandem MS scans was set to 30 s to avoid repetitive scanning of the parent ions.

### Database search

The secondary mass spectrometry data were retrieved using MaxQuant (v1.5.2.8). Retrieval parameter setting: the BioProject number for the database is PRJNA935845 (25518 sequences, *E. coli* genomes ASM886v2 and ASM584v2, and *Klebsiella pneumoniae* genomes ASM24018v2 and ASM383019v1, genes from these genomes were deleted on the comparison), an reverse decoy database was added to calculate the false discovery rate (FDR) caused by random matching, and a common contaminated library was added to the database to eliminate the influence of contaminated proteins in the identification results. The enzyme digestion mode is set to Trypsin/P. The number of missing bits is set to two. The minimum length of the peptide was set to seven amino acid residues. The maximum number of peptide modifications was set to five. The mass error tolerance of primary parent ions of First search and Main search are set to 20 and 5 ppm, respectively, and the mass error tolerance of secondary fragment ions is 0.02 Da. Cysteine alkylation was set as fixed modification, variable modification were methionine oxidation, protein N-terminal acetylation, and deamidation. The quantitative method is set to TMT-6plex, and the FDR for protein identification and PSM identification is set to 1%.

### Bioinformatics analysis

The screening criteria of differential proteins were: D10/D168 ratio ≥1.2 and D10/D168 *p*-value <0.05 as up-regulated proteins; D10/D168 ratio ≤0.83 and D10/D168 *p*-value <0.05 as down regulated proteins. Sequence information for PRJNA935845 were mapped to the NR (NCBI non-redundant protein sequences), NT (NCBI non-redundant nucleotide sequence), SwissProt (a manually annotated and reviewed protein sequence database), KEGG (Kyoto Encyclopedia of Genes and Genomes, version 59), and KOG (Clusters of Eukaryotic Orthologous Groups) database by Blast software (version 2.2.23) with default parameters (under a threshold *E*-value ≤10^−5^) to get the isoform annotations (17). Gene Ontology (GO) annotations and functional classifications were obtained using Blast2GO program (version 2.5.0, *E*-value ≤10^−5^) based on NR annotations (20). InterProScan5 software (version 5.11–51.0) was used to get annotations from InterPro database (21).

Proteins were classified by Gene Ontology (GO) annotation into three categories: biological process, cellular compartment, and molecular function. For each category, a two-tailed Fisher's exact test was employed to test the enrichment of the differentially expressed protein against all identified proteins. The GO with a corrected *p*-value < 0.05 is considered significant. Kyoto Encyclopedia of Genes and Genomes (KEGG) database was used to identify enriched pathways by a two-tailed Fisher's exact test to test the enrichment of the differentially expressed protein against all identified proteins. The pathway with a corrected *p*-value < 0.05 was considered significant. Annotation information of KEGG was obtained by alignment using diamond (v0.9.26.127) software based on KOBAS (V3.0) (containing species sequence information) and KEGG database (sequence corresponding KO information). These pathways were classified into hierarchical categories according to the KEGG website. In the cluster analysis of functional enrichment of differentially expressed proteins, all the categories obtained after enrichment and their *p*-values were first collated, and then filtered for those categories which were at least enriched in one of the clusters with *p*-value <0.05. This filtered *p-*value matrix was transformed by the function *x* = –log_10_ (*p*-value). Finally these *x*-values were *z*-transformed for each functional category. These *z*-scores were then clustered by one-way hierarchical clustering (Euclidean distance, average linkage clustering) in Genesis. Cluster membership were visualized by a heat map using the “heatmap.2” function from the “gplots” R-package.

Construction of protein–protein interaction (PPI) networks was also conducted by using the STRING (v11.0) database with R package “networkD3” tool. All identified protein name identifiers were blast to *Trichomonas vaginalis* (Taxonomy id:5722) and searched against the STRING database version 11.0 for protein–protein interactions. Only interactions between the proteins belonging to the searched data set were selected, thereby excluding external candidates. STRING defines a metric called “confidence score” to define interaction confidence; we fetched all interactions that had a confidence score ≥0.7 (high confidence).

### Targeted protein quantification by LC-PRM/MS analysis

To further validate the protein expression level gained through TMT quantification, additional quantification through parallel reaction monitoring (PRM) analysis was performed (22). Equal amounts of each sample protein were taken for enzymatic lysis, and the volume was adjusted to unity using lysis buffer. The final concentration of 20% TCA was slowly added, vortexed to mix, and precipitated at 4°C for 2 h. Centrifuge at 4,500 g for 5 min, discarded the supernatant and washed the precipitate 2–3 times with pre cooled acetone. After air drying the pellet was added to a final concentration of 200 mM TEAB, the pellet was disrupted by sonication, and trypsin was added at a 1:50 ratio (protease: protein, m/m), and enzymatically digested overnight. Dithiothreitol (DTT, Sigma, HangZhou, China) was added to a final concentration of 5 mm and reduced at 56°C for 30 min. After that, iodoacetamide (IAA, Sigma, HangZhou, China) was added to a final concentration of 11 mm and incubated for 15 min in the dark at room temperature. Peptides were separated after dissolved them in solvent A (0.1% formic acid in 2% acetonitrile). The gradient was comprised of an increase from 7% to 25% solvent B (0.1% formic acid in 90% acetonitrile) over 24 min, 25% to 35% in 8 min, and climbing to 80% in 4 min then holding at 80% for the last 4 min, all at a constant flow rate of 500 nl/min on an EASY-nLC 1000 UPLC system. Then the peptides were injected into the NSI ion source for ionization after separation by the ultra-high-performance liquid phase system and then subjected to Q Exactive^TM^ Plus mass spectrometry for analysis. The ion source voltage was set to 2.1 kV, and both peptide parent ions and their secondary fragments were detected and analyzed using the high-resolution 16/18 Orbitrap. The primary mass spectrometry scan range was set to 437–909 m/z, and the scan resolution was set to 70,000; the secondary mass spectrometry Orbitrap scan resolution was set to 17,500. Data acquisition mode a data-independent acquisition (DIA) program was used with the fragmentation energy of the HCD collision cell set to 27. Generated MS data were processed using skyline (v.3.6) to obtain the signal intensities for individual peptide sequences.

## Results

### Animal inoculation test

**Figure 1:**
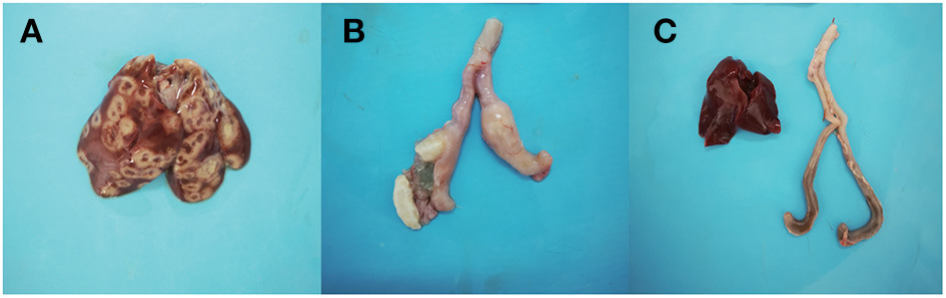
Group D10 liver **(A)**. Group D10 cecum **(B)**. Group D168 liver and cecum **(C)**.

The symptoms after inoculation with the D10 strain in the chickens included depression and diarrhea, with four chickens experiencing symptoms and the morbidity reached 40%. No abnormal symptoms were found in D168 group. In the D10 group, crater-like circular ulcerative necrotic areas appeared on the liver surface of chickens, the cecal wall was thickened and intestinal emboli formed in the ceca (Figures 1A, B). No significant macroscopic lesions were observed in the livers and ceca of D168 group chickens (Figure 1C).

### Tandem mass tag-labeled proteomics analysis

**Figure 2:**
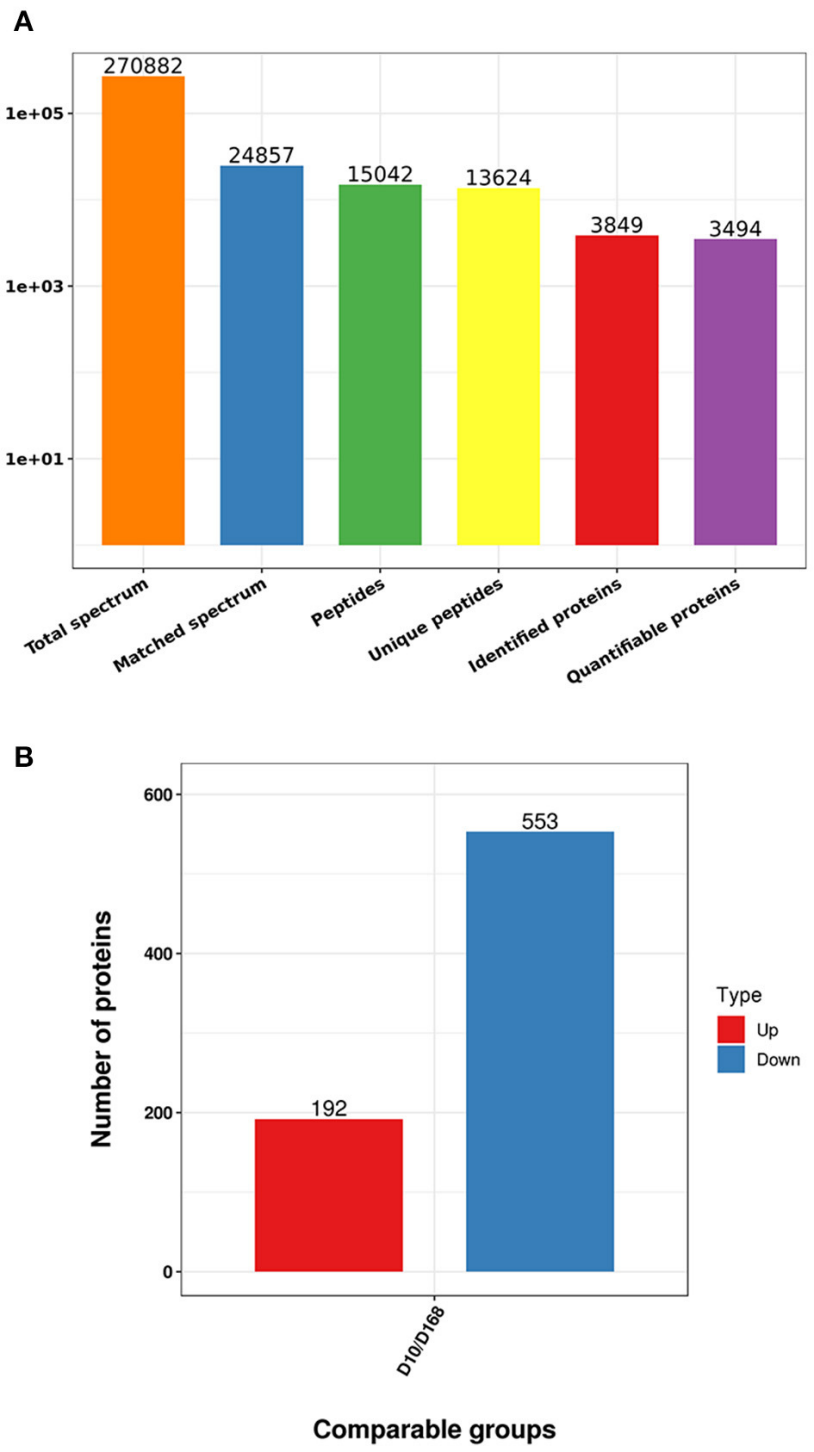
Statistics of mass spectrometry data **(A)**, number of differentially expressed proteins in D10 relative to D168 **(B)**.

Based on TMT labeling and LC-MS/MS approaches, the difference between virulent strain and attenuated strain after continuous passage of *H. meleagridis* were analyzed. From MS analysis, 270,882 spectra were obtained, of which 24,857 effective spectra were available and the utilization rate was 9.2%. A total of 15,042 peptides were identified from spectral analysis, of which specific peptide numbered 13,624. A total of 3,849 proteins were identified, of which 3,494 were quantified (Figure 2A). When *p*-value < 0.05, the variation threshold of significant up-accumulation was set as the variation of differential expression ≥1.2, and the variation threshold of significant down-accumulation was set as the variation threshold ≤0.83. A total of 745 differentially expressed proteins were identified. Relative to D168, there were 192 up-regulated proteins and 553 down-regulated proteins in D10 (Figure 2B, Supplementary Table S1).

### Functional classification of differentially expressed proteins

**Figure 3:**
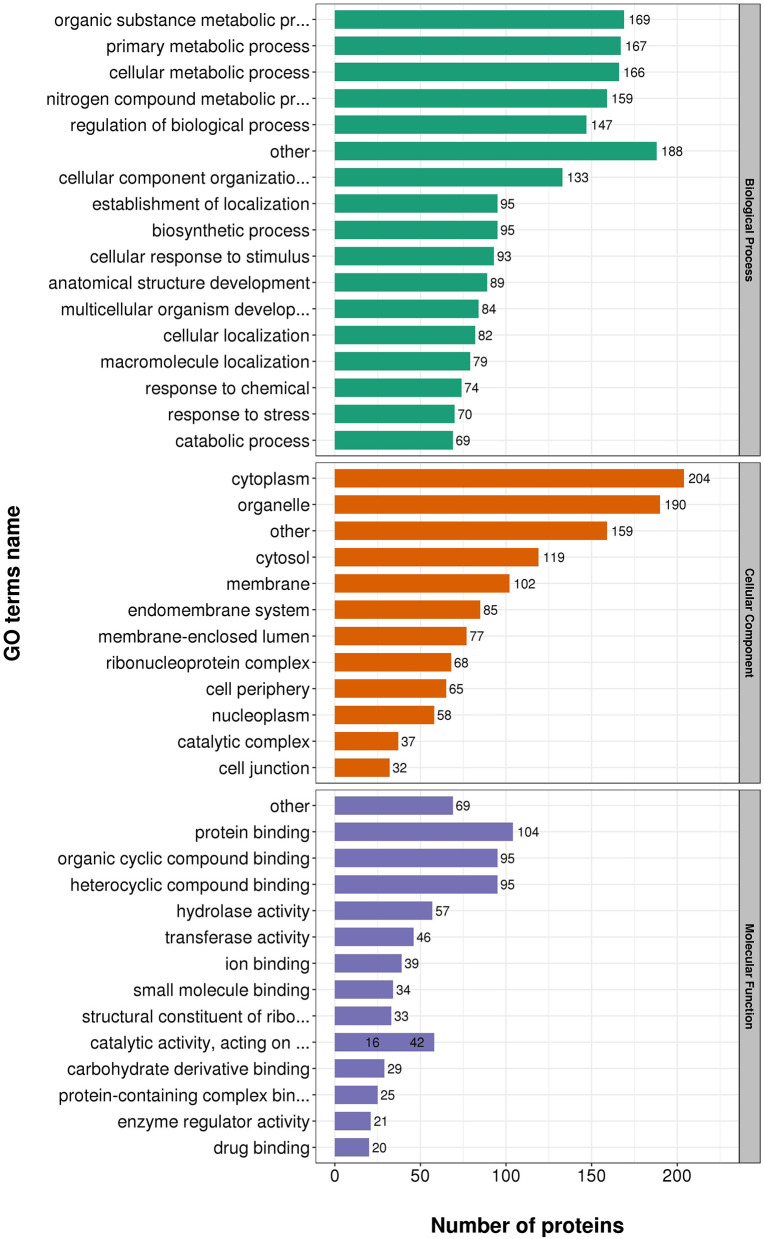
Gene Ontology (GO) analysis of differently expressed proteins based on biological processes, cellular components, and molecular functions.

According to GO annotations, differentially expressed proteins were classified into three categories: Biological Processes, Cellular Components, and Molecular Functions. Biological processes analyses indicated proteins were involved in organic substance metabolic processes, primary metabolic processes, and cellular metabolic processes in both virulent and attenuated *H. meleagridis*. In cell composition analysis, proteins were primarily distributed in cytoplasm, organelles, cytosol, and membrane. In molecular function analysis, proteins were mainly exert the functions of protein binding, organic cyclic compound binding, heterocyclic compound binding (Figure 3).

**Figure 4:**
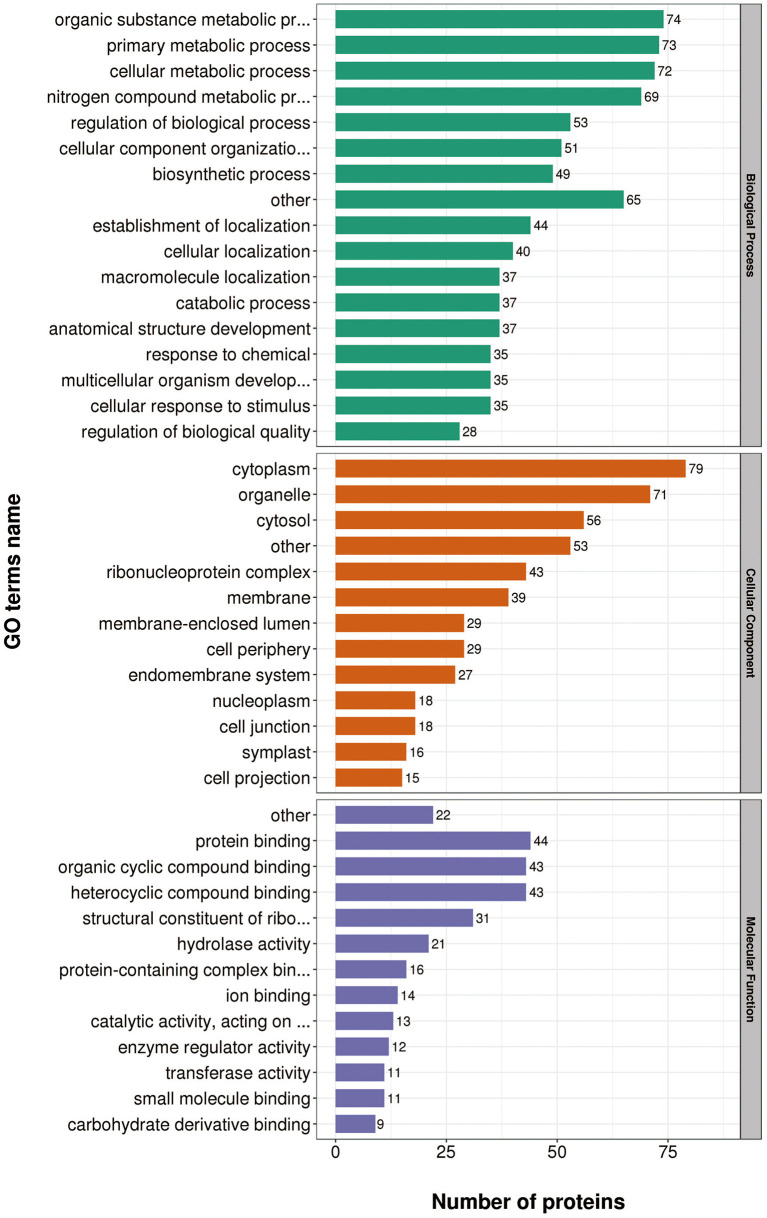
Gene Ontology (GO) analysis of up-regulated proteins in D10 relative to D168.

Gene Ontology (GO) annotations were performed for up-regulated proteins in D10 relative to D168. Biological process analysis showed that proteins mainly participate in the organic substance metabolic process, regulation of biological process, and primary metabolic process. Cellular component analysis revealed that the proteins were mainly distributed in cytoplasm, organelles, and membranes. Molecular functional analysis revealed that the proteins mainly functioned as organic cyclic compound binding, heterocyclic compound binding, structural constituent of ribosome, and hydrolase activity (Figure 4).

**Figure 5:**
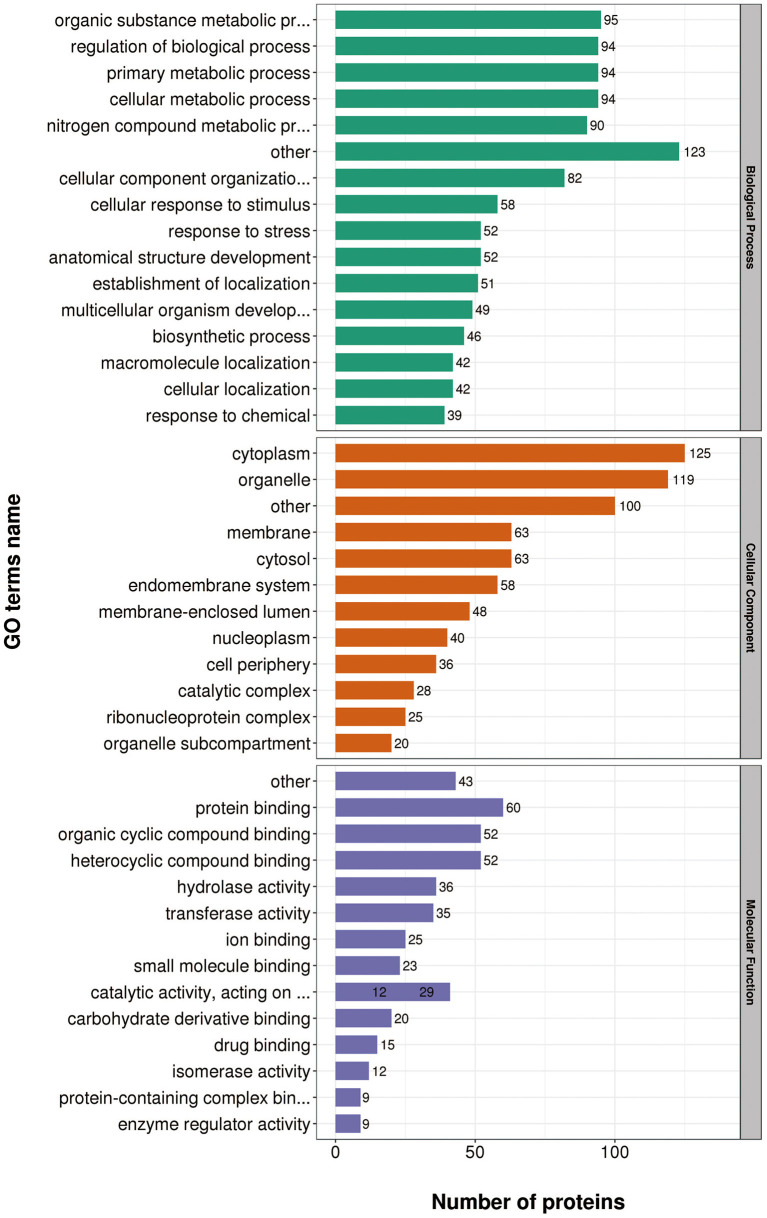
Gene Ontology (GO) analysis of down-regulated proteins in D10 relative to D168.

Gene Ontology (GO) annotations were performed for down-regulated proteins in D10 relative to D168. Biological process analysis showed that proteins mainly participate in the organic substance metabolic process, primary metabolic process, and cellular metabolic process. Cellular component analysis revealed that the proteins were mainly distributed in cytoplasm, organelle, and cytosol. Molecular functional analysis revealed that the proteins mainly functioned in protein binding, organic cyclic compound binding, heterocyclic compound binding, and hydrolase activity (Figure 5).

These data suggested proteins were involved in a variety of biological processes, cellular components, and cellular molecular functions. The ribosome associated functions of the virulent strains were more vigorous and the attenuated strains were more adaptable to the *in vitro* culture environment.

### Gene ontology and Kyoto Encyclopedia of Genes and Genomes cluster analysis of differentially expressed proteins

**Figure 6:**
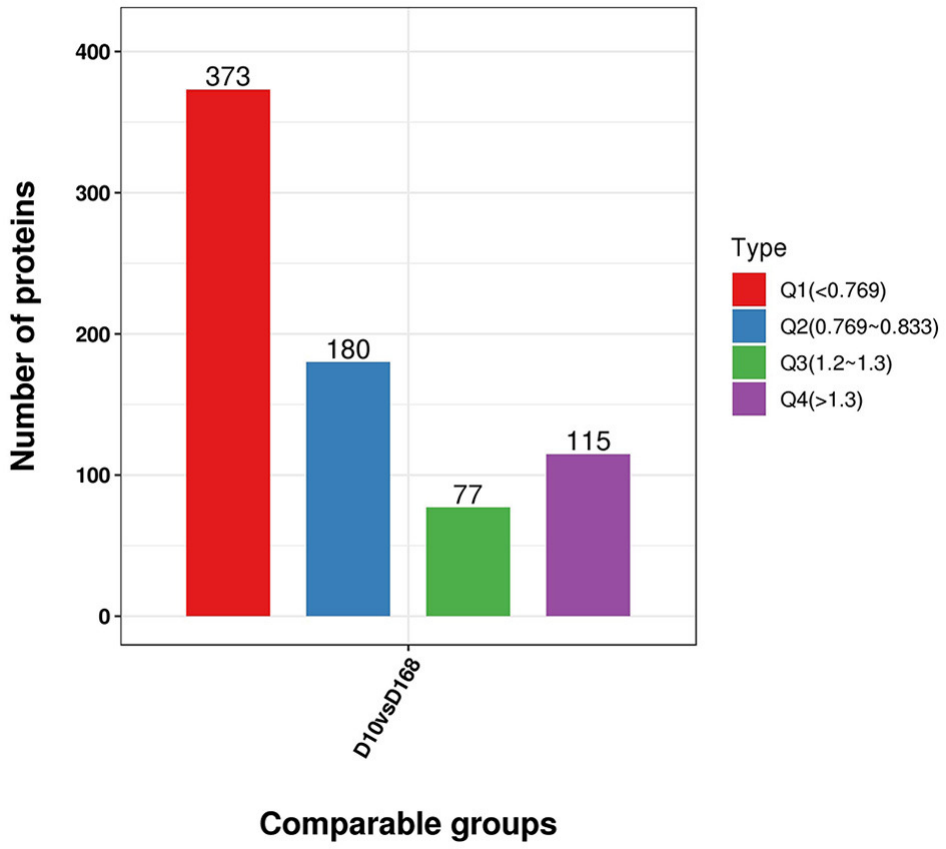
Distribution of differentially expressed proteins in D10 relative to D168 according to the fold change. Q1 was the number of proteins with fold change less than 0.769. Q2 was the number of proteins with fold change between 0.769 and 0.833. Q3 was the number of proteins with fold change between 1.2 and 1.3. Q4 was the number of proteins with fold change above 1.3.

**Figure 7:**
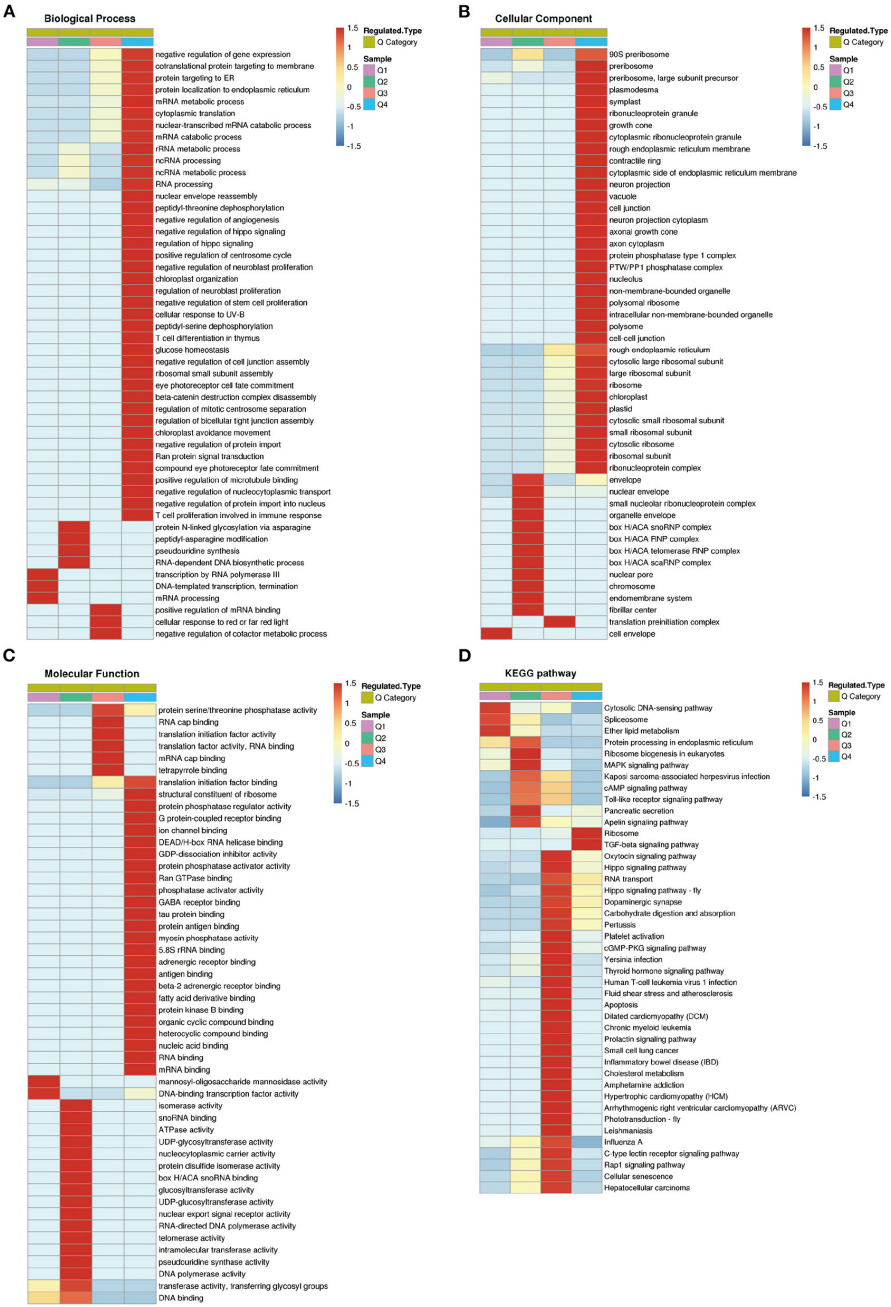
Cluster analysis of differentially expressed proteins based on biological processes, cellular components, molecular functions, and Kyoto Encyclopedia of Genes and Genomes (KEGG) pathways. **(A)** Biological processes. **(B)** Cellular components. **(C)** Molecular functions. **(D)** KEGG pathways.

To further clarify the functional relevance of differential proteins, they were divided into four parts according to the multiple of differences: Q1 < 0.769, 0.769 < Q2< 0.833, 1.2 < Q3< 1.3, Q4 > 1.3 (Figure 6), respectively, enriched the GO and KEGG pathways, and performed cluster analysis. From Figure 6, these analyses showed that for biological processes, proteins in Q1 were primarily involved in transcription by RNA polymerase III, DNA-templated transcription, termination, and mRNA processing. Proteins in Q2 were mainly involved in protein N-linked glycosylation via asparagine, peptidyl-asparagine modification, pseudouridine synthesis, and RNA-dependent DNA biosynthetic process. Proteins in Q3 were mainly involved in positive regulation of mRNA binding, cellular response to red or far red light, and negative regulation of cofactor metabolic process. Proteins in Q4 were mainly involved in negative regulation of gene expression, RNA processing, ribosomal small subunit assembly, and positive regulation of microtubule binding (Figure 7A).

For cellular components, proteins in Q1 were distributed in cell envelope. Proteins in Q2 were mainly distributed in nuclear envelope, organelle envelope, and endomembrane system. Proteins in Q3 were distributed in translation preinitiation complex. Proteins in Q4 were mainly distributed in preribosome, large subunit precursor, plasmodesma, and symplast (Figure 7B).

For molecular functions, proteins in Q1 were concentrated in mannosyl-oligosaccharide mannosidase activity, and DNA-binding transcription factor activity. Proteins in Q2 were mainly concentrated in isomerase activity, ATPase activity, and telomerase activity, etc. Proteins in Q3 were mainly concentrated in RNA cap binding, translation initiation factor activity, and protein serine/threonine phosphatase activity, etc. Proteins in Q4 were mainly concentrated in structural constituent of ribosome, protein phosphatase regulator activity, G protein-coupled receptor binding, and protein phosphatase activator activity (Figure 7C).

KEGG pathway analysis showed that proteins in Q1 were related to cytosolic DNA-sensing pathway, spliceosome, and ether lipid metabolism. Proteins in Q2 were mainly related to ribosome biogenesis in eukaryotes, MAPK signaling pathway, cAMP signaling pathway, and Toll-like receptor signaling pathway. Proteins in Q3 were mainly associated with carbohydrate digestion and absorption, inflammatory bowel disease, cellular senescence, and hepatocellular carcinoma. Proteins in Q4 were associated with ribosome, and TGF-beta signaling pathway (Figure 7D).

### Protein interaction networks

**Figure 8:**
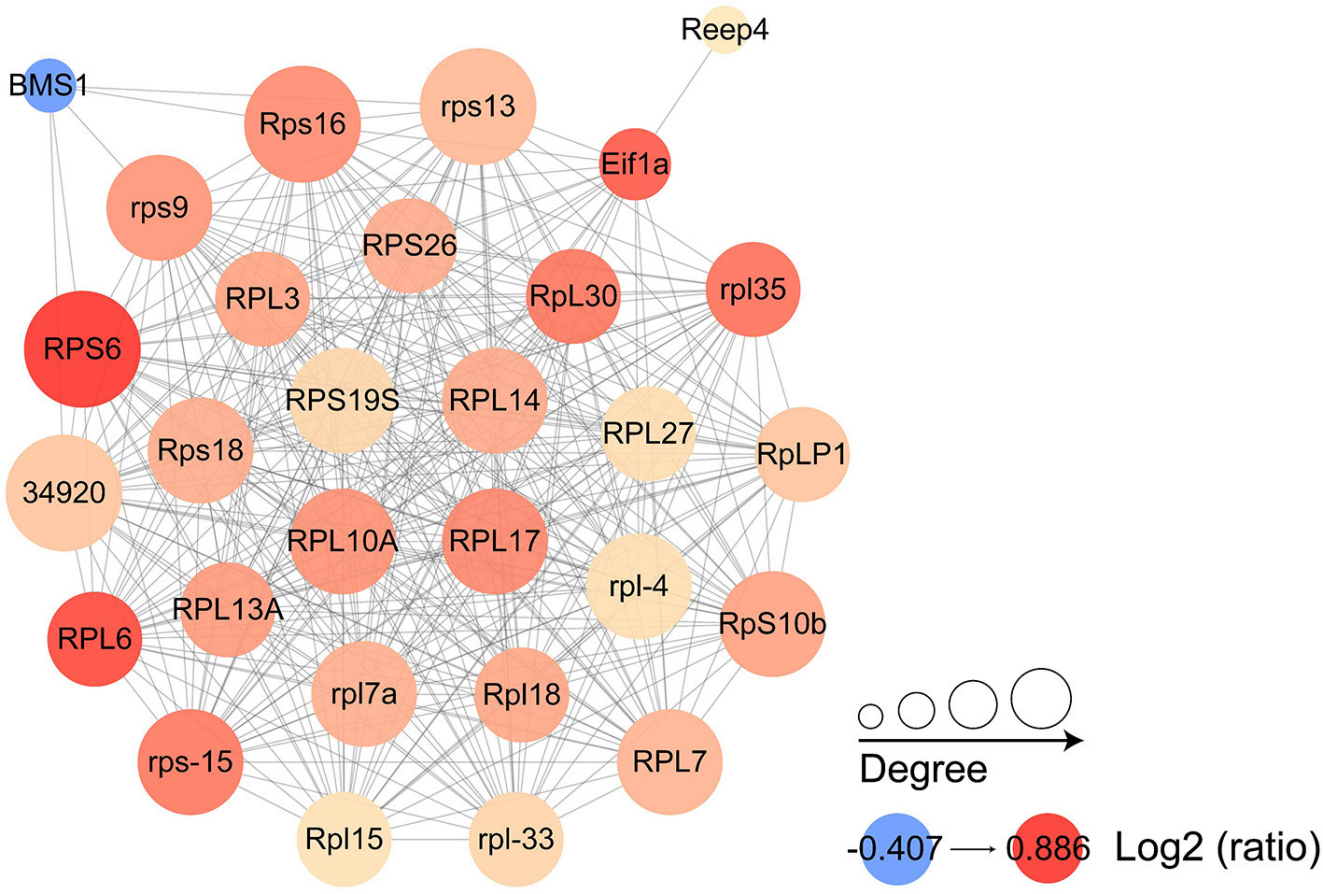
Protein interaction networks of differentially expressed proteins. Each node represents a protein in the graph, each line represents the interaction between proteins, and the larger the circle, the more interactions are involved. The color of the circles shows the log2 ratio, with the darker the color the greater the foldchange.

Proteins were selected for protein interaction network mapping based on pre-existing studies (13, 15, 16, 23), combined with protein function descriptions, including a total of 60 up-regulated proteins and 65 down-regulated proteins. They include virulence-related proteins, such as surface protein BspA-like, digestive cysteine proteinase 2, NADP-dependent malic enzyme; metabolism-related proteins, such as ferredoxin, enolase; cytoskeleton-related proteins, such as actin, alpha-actinin-1; ribosomal proteins, Ras-related proteins, and Ras-like proteins that were abundantly represented in this study (Supplementary Table S2). The results showed that most of the proteins in which the interactions occurred were ribosome associated proteins, which also included eukaryotic translation initiation factors and receptor expression enhancing proteins. The most abundant proteins that undergo interactions are 40S ribosomal protein S19S, 60S ribosomal protein L14, 60S ribosomal protein L17, and 60S ribosomal protein L10a (Figure 8).

### Protein validation by PRM

In order to evaluate the reliability of TMT data, PRM was used to detect the relative levels of ten important functional proteins in the two strains. Seven cysteine related proteins (LCP2, PRPF8, Cts1, KYAT1, cysA, KANK1, metK), two cytoskeleton and movement related proteins (AP3D1, ACTN1), and one cell adhesion related protein (ME1) were selected for PRM analysis. As shown in Table 1, the proteins expression levels were quantified by PRM-MS analysis, which confirmed the protein expression levels obtained by TMT. The results of PRM assay were consistent with those of TMT analysis, indicating that the quantitative results were strong persuasive.

**Table 1:** Comparison of the quantification results between TMT and PRM analyses of the 10 candidate proteins.

**Protein accession**	**Gene name**	**Protein**	**D10/D168 ratio (PRM)**	**D10/D168 ratio (TMT)**
Unigene8555_All_Gene.25220	AP3D1	AP-3 complex subunit delta-1	3.48	1.24
Unigene24108_All_Gene.41566	KYAT1	Kynurenine-oxoglutarate transaminase 1	3.31	1.35
Unigene7145_All_Gene.23035	KANK1	KN motif and ankyrin repeat domain-containing protein 1	2.48	1.25
CL679.Contig1_All_Gene.5371	ME1	NADP-dependent malic enzyme	2.43	1.26
Unigene2364_All_Gene.15201	LCP2	Digestive cysteine proteinase 2	2.26	1.23
Unigene192_All_Gene.12257	cysA	Cystathionine gamma-lyase	3.01	1.22
CL1308.Contig1_All_Gene.8420	metK	S-adenosylmethionine synthase	2.12	1.25
CL707.Contig2_All_Gene.5558	ACTN1	Alpha-actinin-1	2.39	1.35
Unigene13844_All_Gene.31748	PRPF8	Pre-mRNA-processing-splicing factor 8	0.94	0.72
Unigene6832_All_Gene.22514	Ctsl	Cathepsin L1	0.73	0.48

## Discussion

Traditional 2-DE and 2-D-DIGE experimental methods are laborious and difficult to automate, and their dynamic ranges are limited, and proteins are not immediately compatible with mass spectrometry after electrophoretic separation (19). Based on this, the TMT proteomic method, which is more novel, easier to operate and more accurate, and sensitive for peptides detection (19), was chosen and data were obtained for up to 745 differential proteins. Meanwhile, the obtained results were validated using a PRM method that can quantify a set of proteins reproducibly, completely, with high sensitivity and accuracy, ensuring the reliability of the obtained data (18, 22).

The characteristic lesions mainly caused by *H. meleagridis* are crater-like circular ulcerative necrotic areas in the liver and enlargement of the cecum, forming intestinal embolism (2, 4). In the KEGG pathway analysis of the differentially expressed proteins, the up-regulated proteins were indeed associated with inflammatory bowel disease, cellular senescence, and hepatocellular carcinoma pathways. This part of proteins may be responsible for the lesions caused by the virulent strains in the host. In combination with the available reports, *H. meleagridis* virulence factors include adhesion proteins, surface antigens, lectins, cysteine peptidases, GP63-like proteins, phospholipases, pore-forming proteins, and other virulence factors (13). In our study, proteins consistent with these descriptions or functions and up-regulated in virulence strains were indeed identified. The first was surface protein BspA-like. It is a surface protein of Bacteroidales/Spirochaetales that is involved in adhesion to host cells (24). It also mediates host-pathogen interactions and promote cell aggregations (25, 26). BspA has also been found in other eukaryotic pathogens, and in *Entamoeba* they were found to be involved in chemotaxis to tumor necrosis factor (27). In *Trichomonas vaginalis* and *Tetratrichomonas gallinarum*, BspA-like proteins promote parasite adhesion to host cells, suggesting a contribution to pathogenesis (24). It has been shown in pre-existing studies in *H. meleagridis* that more than 80% of BspA-like proteins carry at least one transmembrane helix (28). This suggests that they most likely play a similar role in *H. meleagridis* as in *T. vaginalis* in promoting parasite adhesion to host cells. The next focus was on digestive cysteine proteinase, a protein that has been shown to be involved in pathogenic processes in several pathogens. In *Giardia duodenalis*, cysteine proteases play a role in the pathophysiology of parasitic infection by inducing the degradation and redistribution of the intestinal epithelial cytoskeletal protein villin via myosin light chain kinase (29). Also in *Trichinella spiralis*, cysteine proteases are important for the nutrient acquisition, immune evasion, and invasion in the host (30). In *Entamoeba histolytica* with a closer in origin to *H. meleagridis*, cysteine proteases are important virulence factors that help them to disrupt the host intestinal epithelial barrier functions and circumvent host immune responses (31–33). In connection with the fact that *H. meleagridis* causes severe cecum damage (34), cysteine proteases may play a facilitating role in this process. What was noticed afterwards was actin. Previous studies have shown high expression of actin in virulent strain (15, 16), and this was also true in our study. Actin is an essential component of the cytoskeleton, while it is also involved in many cellular functions, including cell motility, maintenance of cell shape, and transcriptional regulation of polarity (35). In fact, actin is also indirectly involved in the pathogenic process. *Toxoplasma gondii* evolved its own actin cytoskeletal systems for active invasion, and when it invades host cells, it secretes a G-actin binding protein, which is one of its toxins (36, 37). The combination of actin and plasminogen (PLG) is an important step in the activation and maintenance of plasmin. Plasmin degrades the extracellular matrix (ECM) and helps parasites in blood vessels avoiding blood clots (36). In later stage of infection, virulent *H. meleagridis* can metastasize via blood vessels to the liver and other organs, causing more severe damage (38). Considering the various functions of actin, it may play an important role in the pathogenesis of *H. meleagridis*. It was finally noticed that GH family 25 lysozyme presented the highest up-regulation in virulence strain. Lysozyme is a hydrolase of bacterial cell wall peptidoglycan (39). And an important feature of *H. meleagridis* is its complex interaction with bacteria, both *in vivo* and *in vitro* (40). In *T. vaginalis*, which can acquire genes related to the parasite genome by lateral gene transfer from bacteria (41). Combined with the phenomenon that the pathogenicity of *H. meleagridis* decreases gradually with the increase of passage times *in vitro* (10). We speculated that the virulent *H. meleagridis* would acquire partial related virulence genes from the bacteria, but its gradually reduced ability to take up bacterial genes with increasing passage times *in vitro* led to the decline of pathogenicity. However, this still needs further validation.

In addition to the proteins related to virulence factors, another part of up-regulated proteins, which molecular functions concentrated in structural constituent of ribosome, protein phosphatase regulator activity, and KEGG pathway associated with Ribosome, TGF-beta signaling pathway, were equally concerned. The first noted protein was ferredoxin. *H. meleagridis* is a amitochondriate anaerobic protozoa, so pyruvate or malate is further transformed in the hydrogenosomes (42). Ferredoxin is one of the important proteins involved in this process (43). In *T. vaginalis*, changes in the amount of ferredoxin expression correlated with Trichomonas resistance to 5-nitroimidazole drugs (44). The same results were also found in *Giardia lamblia*. Decreased expression and activity of ferredoxin reduced the amount of metronidazole drugs entering the cells, resulting in drug resistance (45). Resistance to metronidazole and nitarsone has also been reported in *H. meleagridis*, but the mechanism of drug resistance has not been deeply studied (46, 47). Given the close relatedness of *H. meleagridis* to *T. vaginalis*, it is reasonable to suspect that the mechanisms underlying resistance to drugs such as metronidazole are similarly proximal and also correlated with changes in the amount of ferredoxin expressed. The second was ribosomal proteins that accounted for 36 of the 192 up-regulated proteins, such as 60S ribosomal protein L6, 40S ribosomal protein S3 and S13. The ribosome, a cornerstone of cell growth and valorization, is composed of ribosomal RNA and ribosomal proteins (48, 49). Ribosomal protein genes are considered to be an interconnected regulator in eukaryotic cells, responding to changes in growth conditions or cellular state (50, 51). In addition to their roles in ribosomal assembly and protein translation, the ribosomal-independent functions of ribosomal proteins are equally important (52). The 60s subunit ribosomal protein of Trypanosomatid has the potential to be a novel pharmaco therapeutic target (53). In visceral Leishmaniasis, the 60S ribosomal protein L6 was suggested to be potentially associated with its resistance to paromomycin (54). Meanwhile its 40S ribosomal protein S12 has the highest sensitivity and specificity as an ELISA assay to detect the target antigen (55). Based on the lack of researches related to ribosomal proteins in *H. meleagridis*, it is believed that it has the potential for further research. The third protein of concern was NADP-dependent malic enzyme. In *T. vaginalis*, all adhesin homologs actually encode hydrogenosomal carbohydrate metabolizing enzymes: alpha-succinyl coenzyme A synthetase (AP-33), beta-succinyl coenzyme A synthetase (AP-51), and malic enzyme (AP-65) (13). Similar to *T. vaginalis*, malic enzyme also functions as an adhesin in *H. meleagridis* (13, 56). Unfortunately, the malic enzyme in this study does not seem to have this function after amino acid alignment. However, it can still be regarded as a drug target. For example, in *Trypanosoma cruzi*, it can be used as a novel drug target because of its importance in maintaining NADPH levels (57).

Furthermore, the down-regulated proteins, which molecular functions concentrated in ATPase activity, telomerase activity and KEGG pathway associated with MAPK signaling pathway, cAMP signaling pathway, were also focused on. These proteins were thought to be relevant to the process of parasites adaptation to *in vitro* culture. The first protein to be noticed was alpha amylase. It is an enzyme widely found in bacteria, fungi, animals, and plants (58). In the *in vitro* culturing system of *H. meleagridis*, rice starch is usually used as one of the additional nutrients that must be added (59). Up-regulation of alpha amylase appeared in both virulent and attenuated strain. Although the one in the virulent strain was identified as pancreatic alpha amylase (60), this still seems to be differentiated from pre-existing reports (23). Such results may be related to the different passage numbers of the attenuated strain and the origin of the *H. meleagridis* strain. The second focus is on ras-like protein 1 and ras-like protein 2. In *Cryptococcus neoformans* and *Saccharomyces cerevisiae*, ras1 and ras2 genes affect the viability of cell growth at all temperatures (61, 62). At the same time, they also have an antagonistic effect in *Candida albicans* (63). In general, after *H. meleagridis* infection, the intestinal tract of animals will provide a relatively stable high temperature and anaerobic environment (64). However, in the process of *in vitro* culture, a passage is required every 2–3 days, resulting in more exposure to oxygen and more frequent temperature changes (10). This may be the root cause of the upregulation of these two proteins in attenuated strain. The last protein noticed in this study was involucrin. Involucrin is a cornified envelope precursor protein expressed by suprabasal terminally differentiated cells (65). Meanwhile, it can also serve as a marker for granular layer keratinocytes (66). Cell keratinization can enhance its resistance to the external environment (67). In addition, involucrin also causes a decrease in the migration speed of the cells (68). This may explain the morphological changes of parasites after serial passage *in vitro* and the phenotypic transition that exhibits higher resilience to the external environment (69).

## Conclusion

This is the first report of protein differences between virulent and attenuated *H. meleagridis* of chicken-origin isolated in China. By using a novel TMT proteomics approach, more differential proteins between the two parasite strains were identified. Most of the proteins reflect the adaptive changing process of the parasite to the environment during *in vitro* culture, which would be helpful to further understand its attenuation mechanism *in vitro*. Whether there are proteins among them that have an effect on their pathogenicity still needs further detailed investigation. In short, this study supplemented the biological data of *H. meleagridis* in China, laying a foundation for further research on this parasite in the future.

## Data availability statement

The datasets presented in this study can be found in online repositories. The names of the repository/repositories and accession number(s) can be found below: https://www.iprox.cn/page/project.html?id=IPX0005472000.

## Ethics statement

The animal study was reviewed and approved by Animal Care and Use Committee of the College of Veterinary Medicine, Yangzhou University.

## Author contributions

Q-GC designed the experiments, conducted most of the experiments, and wrote the manuscript. Y-MZ participated in most of the experiments. CC, SW, and Z-FL created the figures and analyzed the results. D-DL and J-PT helped with the result analysis. Z-FH and J-JX revised the manuscript. All authors contributed to the article and approved the submitted version.
